# The Effect of Physical Activity on Sleep Quality among Older Stroke Survivors: Secondary Analysis from a Randomized Controlled Trial

**DOI:** 10.3390/ijerph192013320

**Published:** 2022-10-15

**Authors:** Srujitha Marupuru, Melanie L. Bell, Michael A. Grandner, Ruth E. Taylor-Piliae

**Affiliations:** 1 College of Pharmacy, The University of Arizona, Tucson, AZ 85721, USA; 2 College of Public Health, The University of Arizona, Tucson, AZ 85721, USA; 3 College of Medicine, The University of Arizona, Tucson, AZ 85721, USA; 4 College of Nursing, The University of Arizona, Tucson, AZ 85721, USA

**Keywords:** chronic stroke, clinical trial, physical activity, sleep quality, stroke rehabilitation

## Abstract

Poor sleep quality constitutes one of the most common difficulties faced by stroke survivors. Physical activity has been shown to improve sleep quality among healthy adults. The study objective was to examine the effect of physical activity on sleep outcomes in community-dwelling stroke survivors previously enrolled in a randomized clinical trial (RCT). Secondary analysis of data collected in the RCT was used to examine the effects of physical activity (PA) on sleep outcomes using the Pittsburgh Sleep Quality Index (PSQI), compared to usual care (controls). Unadjusted and adjusted mixed effects models were used to model changes in sleep quality between groups. At baseline, poor sleep quality (PSQI > 5) was reported by about half of the participants (PA group = 48.5%, n = 47/97; controls = 56.3%, n = 27/48). Results from the unadjusted and adjusted models for sleep quality were similar and showed no statistically significant differences between groups (*p* > 0.05). In the unadjusted model, the difference between groups (change from baseline to 24 weeks) showed that the PA group had better sleep quality than the controls (difference= −1.02 points, 95% CI −2.12, 0.07, *p* = 0.07). In the model adjusted for age, social support, and marital status, the difference between groups (change from baseline to 24 weeks) showed that the PA group had better sleep quality than the controls (difference= −1.07 points, 95% CI −2.19, 0.05, *p* = 0.06). PA did not significantly improve sleep quality in older community-dwelling stroke survivors. Further research is needed to confirm or refute these findings.

## 1. Introduction

Stroke, also known as a cerebrovascular accident, is caused by different acute cerebral circulation disorders (cramps, occlusion or rupture) that restrict blood flow to the brain [1]. According to reported global estimates, approximately 15 million people suffer from a stroke each year, resulting in 5.5 million deaths, with 5 million who cannot live independently because of a disability [2]. Stroke is a leading cause of mortality and disability worldwide and there are substantial economic costs for post-stroke care [3]. Older age and female gender are associated with greater stroke risk. Prevalence of stroke in the United States increases with advancing age in both males and females. The incidence of stroke is likely to continue to escalate because of an expanding population of older Americans [4].

Disabilities following a stroke often cause reduced daily living activities among stroke survivors and lead to negative health outcomes [5]. Older stroke survivors are at higher risk of mortality, poorer functional outcomes, prolonged length of hospital stay, and institutionalization [6]. The most frequent deficiency after a stroke is motor impairment, which can develop as a direct result of the cerebral cortex’s inability to transmit signals, as a gradual process brought on by the damage to the brain, or as a result of muscle atrophy brought on by learned disuse [7,8]. Thus, post-stroke, diminished quality of life and limitations in physical functioning often exist in older stroke survivors.

Sleep difficulties are frequently reported by stroke survivors. Sleep disturbance occurs or worsens after a stroke, and presents in various forms such as sleep apnea, insomnia, or daytime sleepiness [9]. Poor sleep quality can impede stroke rehabilitation, lengthen hospital stay, and influence stroke outcomes and stroke recurrence [10]. On the other hand, regular physical activity and exercise have the potential to positively influence multiple physical and psychosocial domains post-stroke. Physical activity has been shown to improve quality of sleep both in general sleep quality and also other sleep parameters among healthy adults [11]. Among older adults, better sleep quality has been reported after participating in walking [12], or moderate-intensity aerobic and resistance training [13]. In this study, physical activity is defined as body movements produced by the muscles, which require energy expenditure greater than the level of rest [14]. The objective of this study was to examine the effect of a physical activity intervention on sleep quality among older community-dwelling stroke survivors.

## 2. Methods

This study was a secondary analysis of data collected in the “Tai Chi Exercise for Stroke Survivors Study” a prospective four-year randomized clinical trial [15]. 

### 2.1. Study Background and Participants

In the original study, the participants were on average 70 years old, and 3 years post-stroke, with the majority reporting an ischemic stroke (66%), hemiparesis (73%) and male gender (53%) at study enrollment. Details about this study, and recruitment strategies have been previously published [15,16]. Briefly, participants were recruited in cohorts of 12–15 stroke survivors and randomly assigned to Tai Chi exercise, SilverSneakers^®^ exercise or usual care groups, using simple randomization with allocation concealment. Tai Chi is a low-impact, moderate-intensity exercise that combines physical movements with mental concentration, and has been practiced in China over a 1000 years [17]. SilverSneakers^®^ (http://www.silversneakers.com/, accessed on 21 August 2021) is a national fitness program for older adults that offers group-based exercise classes (e.g., muscular strength and range of movement) [18]. Both Tai Chi and SilverSneakers^®^ exercise are low-cost, and safe for persons with chronic diseases or disabilities [18,19]. Participants in the Tai Chi and SilverSneakers^®^ groups attended a one-hour class three-times a week for 12 weeks. Among older adults, Tai Chi and SilverSneakers^®^ are low-moderate intensity forms of physical activity [20,21]. Participants in the usual care (control) group received written materials and resources for participating in community-based physical activity suitable for older adults, along with a phone call once a week for 12 weeks to enquire about their health status. After the 12-week interventions, all participants were followed up for an additional 12 weeks to determine retention of benefit. The 12-week intervention adherence rates in the original study were high (85% overall for all prescribed sessions) [15]. Since the objective of this study was to investigate the effect of physical activity on sleep outcomes in stroke survivors, we combined the Tai Chi and SilverSneakers^®^ groups to form a single physical activity (PA) intervention group for this secondary analysis of data, to compare with usual care (controls). Approval to conduct the study was obtained from the Human Subjects Protection Program at the University of Arizona in Tucson, AZ (approval #0800000257), and written informed consent was obtained from all study participants, in accordance with the principles stated in the Declaration of Helsinki.

### 2.2. Study Measures

Demographic characteristics for age, gender, marital status, employment status and education levels were collected at baseline. Sleep quality was measured at baseline, post-intervention at 12-weeks, and the follow-up assessment at 24-weeks.

#### Pittsburgh Sleep Quality Index (PSQI)

Sleep quality was assessed using the PSQI, a 19-item self-report measure that takes approximately 5–10 min to complete [22]. The PSQI has been successfully used in a variety of patient populations. Psychometric evaluation of the PSQI has established construct, convergent, discriminate and known-groups validity, strong test–retest reliability (r = 0.87), as well as good internal consistency (Cronbach’s alpha = 0.80) [23,24]. The PSQI global score ranges from 0–21, with higher scores indicating poorer sleep quality. The PSQI includes seven components, i.e., subjective quality, latency, duration, efficiency, disturbances, use of medications, and daytime dysfunction (range 0–3, each component), to determine the total global score. A global PSQI score > 5 indicates poor sleepers [22]. In this study, the primary outcome was the difference in the mean change in the PSQI global score from baseline to 24-weeks, between the PA intervention and control groups. The seven sleep components were considered as secondary outcomes.

### 2.3. Statistical Analysis

Descriptive statistics were used to characterize the sample, including means and standard deviations, or medians and interquartile ranges for continuous variables, with frequencies and percentages calculated for categorical variables. Unadjusted and adjusted mixed effects models were used to model sleep quality, as measured by the PSQI. The mixed model for repeated measures, which uses unstructured time and covariance, was used to avoid model misspecification [25], so that terms for time, group, and their interaction was used in the model. Candidate covariates for adjusted models were determined by background knowledge and literature review, and included age, gender (male, female), marital/partnered status (yes/no), income > $50,000 (yes/no), race/ethnicity (white, non-white) and social support as measured by the Multidimensional Scale of Perceived Social Support [26,27]. The final adjusted model included covariates with an absolute value of correlation with sleep quality greater than 0.1. The mixed model for repeated measures accounts for the longitudinal design, is consistent with an intention-to-treat analysis, is robust to missing data [28], and allows for estimating differences between groups and changes within [29,30]. Furthermore, mixed models are more powerful than using *t*-tests [31]. We also undertook similar unadjusted analyses for each of the seven component scores. All analyses were performed using SAS 9.4 (SAS Institute, Cary, NC, USA) with statistical significance set at 0.05.

## 3. Results

### 3.1. Study Sample

**Figure 1:**
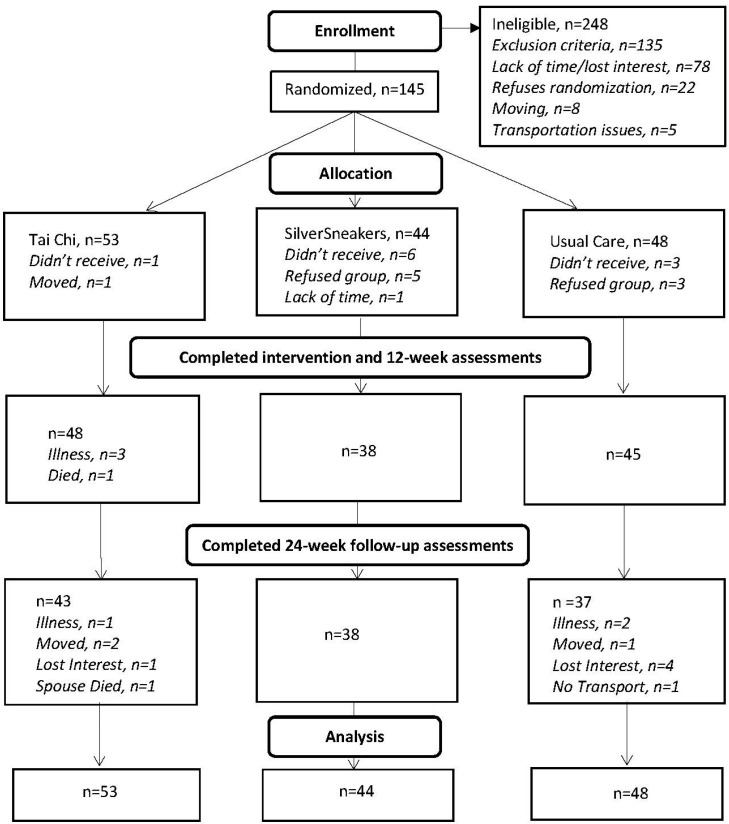
Original Study Flowchart.

The dataset used for this secondary analysis consisted of 145 stroke survivors (PA intervention group, n = 97; control group, n = 48). The recruitment, randomization, and retention flowchart for the original study is shown in Figure 1. 

Study participants were on average 70 ± 10 years old. At baseline, there were no statistically significant differences between groups according to age, gender, income, or sleep quality. However, participants in the PA intervention group were more likely to be married/partner (*p* = 0.005), retired (*p* = 0.04), and white/European-American (*p* = 0.01), than those in the control group. Participants in the control group were more likely to be current smokers (*p* = 0.03), compared to those in the PA intervention group (Table 1).

### 3.2. Sleep Quality Descriptives

Descriptive statistics for these stroke survivors PSQI total and components scores at baseline, 12-weeks post-intervention and at the 24-weeks follow-up assessments are presented in Table 2. PSQI total mean scores > 5 were present at all time points for both the PA intervention and control groups, indicating poor sleep quality. Higher mean scores were observed for the disturbances and daytime dysfunction components, while the medication use component had the lowest mean scores at all time points for both groups.

### 3.3. Sleep Quality Group Comparisons

Results from unadjusted and adjusted models for sleep quality were similar and showed no statistically significant differences between groups (Table 3). The primary outcome of the difference between groups in the change from baseline to 24 weeks showed that the PA intervention group had better sleep quality by 1.02 points (95% CI −2.12, 0.07 for PA—control), but this result did not attain statistical significance (*p* = 0.07). In the model adjusted for age, social support, and marital status, differences between groups in the change from baseline to 24 weeks showed that the PA intervention group had better sleep quality by 1.07 points (95% CI −2.19, 0.05, *p* = 0.06), but this result did not attain statistical significance. Secondary analyses of the sleep quality component scores (See Appendix A for Supplementary data) showed similar results, with only the change in medication use from baseline to 24 weeks as statistically significant (difference = −0.31, 95% CI −0.54, −0.09, *p* = 0.006).

## 4. Discussion

Our secondary analysis of sleep quality outcomes measured by global PSQI scores of older community dwelling stroke survivors found no statistically significant differences in the change in PSQI scores overtime between the PA intervention and control groups. This is an unexpected finding given that meta-analyses of PA interventions indicate significantly better sleep quality and other sleep outcomes post-intervention among healthy adults and those with co-morbid health conditions [32]. Several studies have examined the effect of Tai Chi on sleep quality [33,34], though no studies have examined the effect of SilverSneakers^®^ on sleep quality. To date, only a few studies have examined the effect of Tai Chi on sleep quality among stroke survivors [19,35]. Our results are similar to a pilot randomized clinical trial that examined the effects of Tai Chi on sleep quality in patients with stroke, compared to usual care [35]. Wang and colleagues reported that there was no significant difference in global PSQI scores (*p* = 0.167) overtime between groups. However, our results are in conflict with a different pilot randomized clinical trial conducted among stroke survivors, that reported better sleep quality among those in the Tai Chi intervention (−28.2% change after 12-weeks), compared to usual care (−12.3% change after 12-weeks) [19].

Promotion of physical activity in general is an appropriate aid in the rehabilitation of stroke survivors, to improve sleep quality. However, only a few observational studies have examined the associations between physical activity and sleep quality among stroke survivors [5,36]. In these studies, stroke survivors’ sleep was assessed objectively using accelerometers, though physical activity was not associated with sleep efficiency [36], and there were no significant differences in sleep duration according to gender, stroke type, or hemiparesis side [5]. As with all observational studies, no causal inferences can be determined based on these findings, and thus it is impossible to establish if physical activity improves sleep quality in stroke survivors. Therefore, additional rigorous experimental studies are needed to aid in clinical decision making. In addition, studies should consider characteristics of these stroke survivors, such as whether they have cerebral hemorrhage or cerebral ischemia, different duration of stroke from the first onset, and other confounding factors. To make the outcomes and results of future clinical research more reliable and objective, it will be necessary to apply advanced assessment instruments such as polysomnography or accelerometers, to assess the effect of physical activity interventions on sleep quality and other important sleep outcomes [37].

### Study Strengths and Limitations

One of the major strengths of this study is that data used for this secondary analysis comes from a randomized clinical trial, thus robustness of the data collected can be assured. The study interventions were held at different locations in the community and different times of the day to prevent cross-contamination. Fidelity of the interventions were monitored by an independent study consultant. Study staff assessing the outcome measures were blinded to group assignment. Additionally, an intention to treat analysis was conducted which helps to have unbiased comparisons among groups as any effects of dropouts on random assignment to treatment group is nullified.

However, a number of limitations must be acknowledged. First, is the selective nature of the study population because only community-dwelling stroke survivors uniquely interested in participating in an exercise study may have volunteered. Hence, generalizability of our study findings is limited. Second, the rate of missing data increased over time for both groups, partially due to loss of follow up, which may have influenced the results obtained. Third, the sleep outcomes in our study are self-reported and the subjective nature of the PSQI may reflect inaccurate information. Additional limitations include the absence of pre-stroke sleep habits, stroke severity, location of the stroke, objective sleep data, and quality of life in the analysis. Finally, we did not conduct any power analysis to determine if the sample size was adequate to detect significant, clinically important differences in sleep quality outcomes between groups.

## 5. Conclusions

Poor sleep quality was reported by participants in both the PA intervention and control group at all time points. However, this study found no evidence that physical activity improved sleep outcomes in community-dwelling stroke survivors, as the difference between groups in the change in sleep quality from baseline to the 24-week follow-up assessment was not statistically significant. While physical activity aids stroke recovery, future studies are needed to examine the efficacy of physical activity interventions on sleep quality among community-dwelling stroke survivors. More accurate and robust objective assessments of sleep are needed to characterize the efficacy of physical activity interventions on sleep outcomes in stroke survivors, before widespread recommendations can be made.

## Figures and Tables

**Table 1:** Baseline Characteristics of Older Stroke Survivors According to Group.

Characteristic	Physical Activity Intervention, n = 97	Controls, n = 48	*p*-Value #
Age, mean years (SD)	70.8 (9.9)	68.3 (10.3)	0.16
Women, n (%)	43 (44.3)	25 (52.1)	0.38
Marital Status, n (%)			0.005
Married/partner	64 (66.0)	20 (41.7)	
Single/divorced/widowed	33 (34.0)	28 (58.3)	
Employment Status, n (%)			0.04
Retired	83 (85.6)	33 (68.8)	
Full or Part-Time	5 (5.2)	8 (16.7)	
Unemployed	9 (9.3)	7 (14.6)	
Income, n (%)			0.97
<$50,000 per year	79 (81.4)	35 (72.9)	
≥$50,000 per year	18 (18.6)	13 (27.1)	
Race/Ethnicity, n (%)			0.01
White/European-American	82 (84.5)	32 (66.7)	
Other *	15 (15.5)	16 (33.3)	
Self-Reported Health Problems, n (%)			
Hypertension	71 (73.2)	35 (72.9)	0.97
Dyslipidemia	61 (62.9)	30 (62.5)	0.96
Diabetes	29 (30.0)	11 (22.9)	0.38
Arrhythmia	26 (26.8)	15 (31.3)	0.58
Major Depression	15 (15.5)	9 (18.8)	0.62
Asthma	11 (11.3)	7 (14.6)	0.58
Chronic Heart Failure	11 (11.3)	11 (23.4)	0.06
Previous Myocardial Infarction	15 (15.5)	7 (14.6)	0.88
Current Smoker	4 (4.1)	7 (14.6)	0.03
Sleep Quality			
PSQI score, median (range = 0–21)	6.2 (3–8)	7.3 (4–11.5)	0.13
Poor Sleep Quality (PSQI > 5), n (%)	47 (48.5)	27 (56.3)	0.38
Sleep Time, mean hours/day (SD)	7.5 (1.5)	7.2 (1.9)	0.25

# two sample *t*-test for continuous variables, chi-square test for categorical variables; * includes American Indian/Alaskan Native, Asian/Asian-American, black/African American, Latino/Mexican-American, Middle-Eastern, Native Hawaiian/Pacific Islander, other; PSQI = Pittsburgh Sleep Quality Index.

**Table 2:** Sleep Quality Scores of Older Stroke Survivors Overtime.

	Baseline		12-Weeks		24-Weeks	
	Physical Activity, n = 94	Controls, n = 48	Physical Activity, n = 85	Controls, n = 45	Physical Activity, n = 80	Controls, n = 38
PSQI total score, mean (SD)	5.69 (3.04)	6.42 (4.11)	5.88 (3.54)	5.82 (3.73)	6.03 (3.45)	5.71 (3.70)
PSQI component scores, mean (SD)						
subjective quality	0.94 (1.27)	0.81 (1.25)	0.88 (1.27)	0.84 (1.26)	0.93 (1.27)	0.74 (1.11)
latency	0.94 (0.89)	0.94 (0.89)	1.04 (0.85)	0.82 (0.91)	1.06 (0.90)	0.89 (0.89)
duration	0.41 (0.76)	0.65 (1.06)	0.47 (0.88)	0.40 (0.75)	0.44 (0.82)	0.47 (0.95)
efficiency	0.70 (0.97)	0.90 (1.17)	0.80 (1.09)	0.76 (1.00)	0.70 (1.00)	0.82 (1.01)
disturbances	1.34 (0.59)	1.42 (0.65)	1.33 (0.54)	1.49 (0.59)	1.45 (0.61)	1.37 (0.63)
medication use	0.22 (0.58)	0.35 (0.76)	0.15 (0.50)	0.33 (0.77)	0.29 (0.68)	0.13 (0.34)
daytime dysfunction	1.27 (0.81)	1.33 (0.95)	1.24 (0.80)	1.18 (0.78)	1.16 (0.82)	1.29 (0.96)

Abbreviations: PSQI = Pittsburgh Sleep Quality Index; total score range = 0–21; higher scores indicate worse sleep quality; possible range for component scores = 0–3.

**Table 3:** Mixed Model Estimates of Sleep Quality Among Older Stroke Survivors.

Sleep Quality Scores (PSQI)	Least Squares Means (SE) Physical Activity (n = 97)	Least Squares Means (SE) Control (n = 48)	Unadjusted Mean Difference (95% CI)	*p*-Value	Adjusted ^a^ Mean Difference (95% CI)	*p*-Value
Baseline	5.67 (0.35)	6.42 (0.50)	−0.74 (−1.94, 0.46)	0.22	−0.41 (−1.65, 0.82)	0.51
12 weeks	5.88 (0.38)	5.79 (0.52)	0.09 (−1.18, 1.36)	0.89	0.32 (−0.95, 1.59)	0.62
24 weeks	6.03 (0.38)	5.75 (0.55)	0.28 (−1.04, 1.60)	0.67	0.66 (−0.73, 2.04)	0.35
Baseline-12 weeks	−0.20 (0.31)	0.63 (0.43)	−0.83 (−1.89, 0.23)	0.12	−0.73 (−1.72, 0.25)	0.14
Baseline-24 weeks	−0.36 (0.32)	0.66 (0.45)	−1.02 (−2.12, 0.07)	0.07	−1.07 (−2.19, 0.05)	0.06

Abbreviations: CI = Confidence Interval, PSQI = Pittsburgh Sleep Quality Index. total score range = 0–21; higher scores indicate worse sleep quality. Estimates are Physical Activity Intervention—Controls. ^a^ Adjusted for age, social support, and marital status.

## Data Availability

The data presented in this study are available on request from the corresponding author. The data are not publicly available for privacy reasons.

## Appendix A. Mixed Model Estimates of the Seven Components of the Pittsburgh Sleep Quality Index

**Table A1:** Subjective Quality Component.

Sleep Quality Scores (PSQI)	Least Squares Means (SE) Physical Activity (n = 97)	Least Squares Means (SE) Control (n = 48)	Unadjusted Mean Difference (95% CI)	*p*-Value
Baseline	0.94 (0.13)	0.81 (0.18)	0.13 (−0.31, 0.57)	0.57
12 weeks	0.91 (0.13)	0.82 (0.18)	0.10 (−0.35, 0.54)	0.67
24 weeks	0.91 (0.13)	0.72 (0.18)	0.19 (−0.25, 0.63)	0.40
Baseline-12 weeks	0.02 (0.09)	−0.01 (0.13)	0.03 (−0.28, 0.34)	0.84
Baseline-24 weeks	0.02 (0.11)	0.09 (0.15)	−0.06 (−0.43, 0.30)	0.73

Abbreviations: CI = Confidence Interval, PSQI = Pittsburgh Sleep Quality Index, SE = standard error. Differences Are Physical Activity Intervention—Control.

**Table A2:** Latency Component.

Sleep Quality Scores (PSQI)	Least Squares Means (SE) Physical Activity (n = 97)	Least Squares Means (SE) Control (n = 48)	Unadjusted Mean Difference (95% CI)	*p*-Value
Baseline	0.94 (0.09)	0.96 (0.13)	−0.02 (−0.34, 0.30)	0.90
12 weeks	1.03 (0.09)	0.81 (0.13)	0.22 (−0.09, 0.53)	0.16
24 weeks	1.05 (0.10)	0.92 (0.14)	0.13 (−0.21, 0.47)	0.45
Baseline-12 weeks	−0.10 (0.09)	0.15 (0.13)	−0.24 (−0.55, 0.07)	0.12
Baseline-24 weeks	−0.11 (0.10)	0.04 (0.14)	−0.15 (−0.49, 0.18)	0.37

Abbreviations: CI = Confidence Interval, PSQI = Pittsburgh Sleep Quality Index, SE = standard error. Differences Are Physical Activity Intervention—Control.

**Table A3:** Duration Component.

Sleep Quality Scores (PSQI)	Least Squares Means (SE) Physical Activity (n = 97)	Least Squares Means (SE) Control (n = 48)	Unadjusted Mean Difference (95% CI)	*p*-Value
Baseline	0.41 (0.09)	0.65 (0.13)	−0.24 (−0.54, 0.07)	0.12
12 weeks	0.45 (0.09)	0.39 (0.12)	0.06 (−0.24, 0.36)	0.69
24 weeks	0.42 (0.09)	0.49 (0.13)	−0.07 (−0.39, 0.25)	0.68
Baseline-12 weeks	−0.05 (0.09)	0.26 (0.13)	−0.30 (−0.62, 0.02)	0.06
Baseline-24 weeks	−0.02 (0.09)	0.15 (0.12)	−0.17 (−0.47, 0.12)	0.25

Abbreviations: CI = Confidence Interval, PSQI = Pittsburgh Sleep Quality Index, SE = standard error. Differences Are Physical Activity Intervention—Control.

**Table A4:** Efficiency Component.

Sleep Quality Scores (PSQI)	Least Squares Means (SE) Physical Activity (n = 97)	Least Squares Means (SE) Control (n = 48)	Unadjusted Mean Difference (95% CI)	*p*-Value
Baseline	0.70 (0.11)	0.90 (0.15)	−0.19 (−0.56, 0.17)	0.29
12 weeks	0.80 (0.11)	0.75 (0.16)	0.05 (−0.33, 0.43)	0.80
24 weeks	0.70 (0.11)	0.85 (0.16)	−0.15 (−0.54, 0.23)	0.43
Baseline-12 weeks	−0.10 (0.12)	0.15 (0.16)	−0.24 (−0.64, 0.15)	0.22
Baseline-24 weeks	0.00 (0.12)	0.04 (0.18)	−0.04 (−0.47, 0.39)	0.85

Abbreviations: CI = Confidence Interval, PSQI = Pittsburgh Sleep Quality Index, SE = standard error. Differences Are Physical Activity Intervention—Control.

**Table A5:** Disturbances Component.

Sleep Quality Scores (PSQI)	Least Squares Means (SE) Physical Activity (n = 97)	Least Squares Means (SE) Control (n = 48)	Unadjusted Mean Difference (95% CI)	*p*-Value
Baseline	1.34 (0.06)	1.42 (0.09)	−0.08 (−0.29, 0.14)	0.48
12 weeks	1.34 (0.06)	1.50 (0.08)	−0.15 (−0.35, 0.05)	0.14
24 weeks	1.46 (0.07)	1.39 (0.10)	0.07 (−0.17, 0.30)	0.58
Baseline-12 weeks	0.00 (0.07)	−0.08 (0.09)	0.07 (−0.15, 0.30)	0.51
Baseline-24 weeks	−0.12 (0.07)	0.02 (0.10)	−0.14 (−0.38, 0.10)	0.24

Abbreviations: CI = Confidence Interval, PSQI = Pittsburgh Sleep Quality Index, SE = standard error. Differences Are Physical Activity Intervention—Control.

**Table A6:** Medication Use Component.

Sleep Quality Scores (PSQI)	Least Squares Means (SE) Physical Activity (n = 97)	Least Squares Means (SE) Control (n = 48)	Unadjusted Mean Difference (95% CI)	*p*-Value
Baseline	0.22 (0.07)	0.35 (0.09)	−0.14 (−0.36, 0.09)	0.23
12 weeks	0.17 (0.07)	0.34 (0.09)	−0.18 (−0.40, 0.04)	0.12
24 weeks	0.32 (0.07)	0.14 (0.10)	0.18 (−0.06, 0.41)	0.14
Baseline-12 weeks	0.05 (0.06)	0.01 (0.08)	0.04 (−0.18, 0.25)	0.72
Baseline-24 weeks	−0.10 (0.06)	0.21 (0.09)	−0.31 (−0.54, −0.09)	0.01

Abbreviations: CI = Confidence Interval, PSQI = Pittsburgh Sleep Quality Index, SE = standard error. Differences Are Physical Activity Intervention—Control.

**Table A7:** Daytime Dysfunction Component.

Sleep Quality Scores (PSQI)	Least Squares Means (SE) Physical Activity (n = 97)	Least Squares Means (SE) Control (n = 48)	Unadjusted Mean Difference (95% CI)	*p*-Value
Baseline	1.27 (0.09)	1.33 (0.12)	−0.07 (−0.37, 0.23)	0.67
12 weeks	1.25 (0.08)	1.18 (0.12)	0.07 (−0.21, 0.36)	0.62
24 weeks	1.19 (0.10)	1.27 (0.14)	−0.09 (−0.42, 0.25)	0.61
Baseline-12 weeks	0.02 (0.09)	0.16 (0.12)	−0.14 (−0.44, 0.17)	0.38
Baseline-24 weeks	0.08 (0.10)	0.06 (0.14)	0.02 (−0.32, 0.36)	0.91

Abbreviations: CI = Confidence Interval, PSQI = Pittsburgh Sleep Quality Index, SE = standard error. Differences Are Physical Activity Intervention—Control.

## References

[1] Chandra A., Stone C.R., Du X., Li W.A., Huber M., Bremer R., Geng X., Ding Y. (2017). The cerebral circulation and cerebrovascular disease III: Stroke. Brain Circ..

[2] Collaborators G.B.D.S. (2019). Global, regional, and national burden of stroke, 1990–2016: A systematic analysis for the Global Burden of Disease Study 2016. Lancet Neurol..

[3] Rajsic S., Gothe H., Borba H.H., Sroczynski G., Vujicic J., Toell T., Siebert U. (2019). Economic burden of stroke: A systematic review on post-stroke care. Eur. J. Health Econ..

[4] Virani S.S., Alonso A., Aparicio H.J., Benjamin E.J., Bittencourt M.S., Callaway C.W., Carson A.P., Chamberlain A.M., Cheng S., Delling F.N. (2021). Heart Disease and Stroke Statistics-2021 Update: A Report From the American Heart Association. Circulation.

[5] Ezeugwu V.E., Manns P.J. (2017). Sleep Duration, Sedentary Behavior, Physical Activity, and Quality of Life after Inpatient Stroke Rehabilitation. J. Stroke Cerebrovasc. Dis..

[6] Lui S.K., Nguyen M.H. (2018). Elderly Stroke Rehabilitation: Overcoming the Complications and Its Associated Challenges. Curr. Gerontol. Geriatr. Res..

[7] Mutai H., Furukawa T., Araki K., Misawa K., Hanihara T. (2013). Long-term outcome in stroke survivors after discharge from a convalescent rehabilitation ward. Psychiatry Clin. Neurosci..

[8] Tang W.K., Lau C.G., Mok V., Ungvari G.S., Wong K.S. (2014). Apathy and health-related quality of life in stroke. Arch. Phys. Med. Rehabil..

[9] Khot S.P., Morgenstern L.B. (2019). Sleep and Stroke. Stroke.

[10] Iddagoda M.T., Inderjeeth C.A., Chan K., Raymond W.D. (2020). Post-stroke sleep disturbances and rehabilitation outcomes: A prospective cohort study. Intern. Med. J..

[11] Kredlow M.A., Capozzoli M.C., Hearon B.A., Calkins A.W., Otto M.W. (2015). The effects of physical activity on sleep: A meta-analytic review. J. Behav. Med..

[12] Cheng H.P., Chen C.H., Lin H.S., Wang J.J., Yen M. (2022). Relationship between Walking Activity and Sleep Quality among Community-Dwelling Older Adults. J. Community Health Nurs..

[13] Zhou Y., Wu W., Zou Y., Huang W., Lin S., Ye J., Lan Y. (2022). Benefits of different combinations of aerobic and resistance exercise for improving plasma glucose and lipid metabolism and sleep quality among elderly patients with metabolic syndrome: A randomized controlled trial. Endocr. J..

[14] Caspersen C.J., Powell K.E., Christenson G.M. (1985). Physical activity, exercise, and physical fitness: Definitions and distinctions for health-related research. Public Health Rep..

[15] Taylor-Piliae R.E., Hoke T.M., Hepworth J.T., Latt L.D., Najafi B., Coull B.M. (2014). Effect of Tai Chi on physical function, fall rates and quality of life among older stroke survivors. Arch. Phys. Med. Rehabil..

[16] Taylor-Piliae R.E., Boros D., Coull B.M. (2014). Strategies to improve recruitment and retention of older stroke survivors to a randomized clinical exercise trial. J. Stroke Cerebrovasc. Dis..

[17] Lan C., Chen S.Y., Lai J.S., Wong A.M. (2013). Tai chi chuan in medicine and health promotion. Evid. Based Complement. Alternat. Med..

[18] Nguyen H.Q., Ackermann R.T., Maciejewski M., Berke E., Patrick M., Williams B., LoGerfo J.P. (2008). Managed-Medicare health club benefit and reduced health care costs among older adults. Prev. Chronic Dis..

[19] Taylor-Piliae R.E., Coull B.M. (2012). Community-based Yang-style Tai Chi is safe and feasible in chronic stroke: A pilot study. Clin Rehabil..

[20] Lan C., Chen S.Y., Lai J.S. (2004). Relative exercise intensity of Tai Chi Chuan is similar in different ages and gender. Am. J. Chin. Med..

[21] Ainsworth B.E., Haskell W.L., Herrmann S.D., Meckes N., Bassett D.R., Tudor-Locke C., Greer J.L., Vezina J., Whitt-Glover M.C., Leon A.S. (2011). 2011 Compendium of Physical Activities: A second update of codes and MET values. Med. Sci. Sports Exerc.

[22] Buysse D.J., Reynolds C.F., Monk T.H., Berman S.R., Kupfer D.J. (1989). The Pittsburgh Sleep Quality Index: A new instrument for psychiatric practice and research. Psychiatry Res..

[23] Carpenter J.S., Andrykowski M.A. (1998). Psychometric evaluation of the Pittsburgh Sleep Quality Index. J. Psychosom. Res..

[24] Backhaus J., Junghanns K., Broocks A., Riemann D., Hohagen F. (2002). Test-retest reliability and validity of the Pittsburgh Sleep Quality Index in primary insomnia. J. Psychosom. Res..

[25] Mallinckrod† C.H., Lane P.W., Schnell D., Peng Y., Mancuso J.P. (2008). Recommendations for the Primary Analysis of Continuous Endpoints in Longitudinal Clinical Trials. Drug Inf. J..

[26] Zimet G.D., Dahlem N.W., Zimet S.G., Farley G.K. (1988). The Multidimensional Scale of Perceived Social Support. J. Personal. Assess..

[27] Zimet G.D., Powell S.S., Farley G.K., Werkman S., Berkoff K.A. (1990). Psychometric characteristics of the Multidimensional Scale of Perceived Social Support. J. Personal. Assess..

[28] Bell M.L., Fairclough D.L. (2014). Practical and statistical issues in missing data for longitudinal patient-reported outcomes. Stat. Methods Med. Res..

[29] Molenberghs G., Thijs H., Jansen I., Beunckens C., Kenward M.G., Mallinckrodt C., Carroll R.J. (2004). Analyzing incomplete longitudinal clinical trial data. Biostatistics.

[30] Mallinckrodt C., Lipkovich I. (2016). Analyzing Longitudinal Clinical Trial Data: A Practical Guide.

[31] Ashbeck E.L., Bell M.L. (2016). Single time point comparisons in longitudinal randomized controlled trials: Power and bias in the presence of missing data. BMC Med. Res. Methodol..

[32] Amiri S., Hasani J., Satkin M. (2021). Effect of exercise training on improving sleep disturbances: A systematic review and meta-analysis of randomized control trials. Sleep Med..

[33] Du S., Dong J., Zhang H., Jin S., Xu G., Liu Z., Chen L., Yin H., Sun Z. (2015). Taichi exercise for self-rated sleep quality in older people: A systematic review and meta-analysis. Int. J. Nurs. Stud..

[34] Li H., Chen J., Xu G., Duan Y., Huang D., Tang C., Liu J. (2020). The Effect of Tai Chi for Improving Sleep Quality: A Systematic Review and Meta-analysis. J. Affect. Disord..

[35] Wang W., Sawada M., Noriyama Y., Arita K., Ota T., Sadamatsu M., Kiyotou R., Hirai M., Kishimoto T. (2010). Tai Chi exercise versus rehabilitation for the elderly with cerebral vascular disorder: A single-blinded randomized controlled trial. Psychogeriatrics.

[36] Shepherd A.I., Pulsford R., Poltawski L., Forster A., Taylor R.S., Spencer A., Hollands L., James M., Allison R., Norris M. (2018). Physical activity, sleep, and fatigue in community dwelling Stroke Survivors. Sci. Rep..

[37] Grandner M.A., Rosenberger M.E., Grandner M.A. (2019). Actigraphic sleep tracking and wearables: Historical context, scientific applications and guidelines, limitations, and considerations for commercial sleep devices. Sleep and Health.

